# The Effect of Heavy Fe-Doping on 3D Growth Mode and Fe Diffusion in GaN for High Power HEMT Application

**DOI:** 10.3390/ma15062058

**Published:** 2022-03-10

**Authors:** Jin-Ji Dai, Thi Thu Mai, Umeshwar Reddy Nallasani, Shao-Chien Chang, Hsin-I Hsiao, Ssu-Kuan Wu, Cheng-Wei Liu, Hua-Chiang Wen, Wu-Ching Chou, Chieh-Piao Wang, Luc Huy Hoang

**Affiliations:** 1 Department of Electrophysics, National Yang Ming Chiao Tung University, Hsinchu 30010, Taiwan; jinjidai@gmail.com (J.-J.D.); maithu1589@gmail.com (T.T.M.); umeshnalasani@gmail.com (U.R.N.); brian4656033@gmail.com (S.-C.C.); hsinisandy@gmail.com (H.-I.H.); wusykuann@gmail.com (S.-K.W.); william798424@gmail.com (C.-W.L.); a091316104@gmail.com (H.-C.W.); 2 Technology Development Division, Episil-Precision Inc., Hsinchu 30010, Taiwan; cp.wang@epi.episil.com; 3 Faculty of Physics, Hanoi National University of Education, 136 Xuan Thuy, Cau Giay, Hanoi 10000, Vietnam

**Keywords:** GaN power HEMT, MOCVD, Fe doping, nano-mask, 3D growth, diffusion

## Abstract

The high electron mobility transistor (HEMT) structures on Si (111) substrates were fabricated with heavily Fe-doped GaN buffer layers by metalorganic chemical vapor deposition (MOCVD). The heavy Fe concentrations employed for the purpose of highly insulating buffer resulted in Fe segregation and 3D island growth, which played the role of a nano-mask. The in situ reflectance measurements revealed a transition from 2D to 3D growth mode during the growth of a heavily Fe-doped GaN:Fe layer. The 3D growth mode of Fe nano-mask can effectively annihilate edge-type threading dislocations and improve transfer properties in the channel layer, and consequently decrease the vertical leakage current by one order of magnitude for the applied voltage of 1000 V. Moreover, the employment of GaN:C film on GaN:Fe buffer can further reduce the buffer leakage-current and effectively suppress Fe diffusion.

## 1. Introduction

GaN-based high electron mobility transistors (HEMTs) on Si substrate have received tremendous attention in recent years due to their high breakdown field, high saturation velocity, and low cost in the application of high-voltage and high-frequency devices [1,2]. However, achieving high breakdown voltage (V_BD_) and low dynamic on-resistance (R_ON_) for the GaN HEMTs remains a challenge [1,3]. A highly insulating buffer with good crystalline quality is required to suppress the leakage current for the GaN-on-Si HEMTs operating at high voltage. The un-doped GaN (u-GaN) on Si structures typically exhibits *n*-type conductivity due to the existence of intrinsic defects, such as high threading dislocation (TD) densities of about 10^9^–10^10^ cm^−2^ resulted from the lattice and thermal expansion mismatch, nitrogen vacancies (V_N_), residual silicon and oxygen impurities [4]. As a result, a comprehensive study to mitigate buffer TD densities and compensate *n*-type defects is highly essential. The purpose of this work is to develop three-dimensional (3D) growth techniques and acceptor doping to reduce TD densities and compensate the intrinsic *n*-type defects, respectively.

There are several methods to achieve 3D growth, such as the epitaxial lateral overgrowth (ELOG) technique [5] either with nano-mask [6,7,8] or without nano-mask [9]. In our previous study, the bulk TD density was mitigated by the insertion of SiN_x_ nano-mask in the low temperature (LT) buffer [6]. In the case of *n*-type defect compensation, the most common approach is using carbon or iron doping to form deep acceptors [10,11,12,13,14]. However, the deep-level traps created by carbon doping result in a parasitic effect on the captured electrons. This effect increases the dynamic on-resistance (R_ON_) and switching losses, which degrades the reliability of the devices [15,16,17,18]. The alternative Fe-doped buffer shows minimum degradation on current collapse phenomena because the acceptor energy level of Fe-doped buffer is shallower than that of the C-doped buffer layer [18,19,20]. Therefore, the GaN HEMT with Fe-doping buffer is currently the predominant structure to meet industry standards for high-frequency power devices.

In general, heavily Fe-doped GaN layers can achieve a very high sheet resistance of 10^10^ ohm/sq [21,22,23]. However, heavy Fe doping for providing higher acceptor concentrations to compensate *n*-type defects encountered problems of Fe segregation and diffusion. The Fe segregation propagates and accumulates on the surface during growth, deteriorating surface morphology and crystalline quality [10,24]. Moreover, these Fe atoms tend to diffuse into the GaN channel and cause the capture of conducting electrons during the on/off device switching operation, further resulting in current collapse and high R_ON_ [23,25]. Several groups proposed different approaches for solving this Fe diffusion issue [10,25,26,27]. A u-GaN buffer layer was inserted between the GaN channel and the Fe-doped buffer to reduce the effect of Fe diffusion and avoid impact on device characteristics [10]. Tetsuro Ishiguro et al. [26] applied compressive strain in GaN layers to effectively suppress Fe diffusion. Furthermore, Leone et al. [27] demonstrated a limiting mechanism for Fe diffusion with the benefit of compressive stress produced in heavily C-doped GaN.

In this study, for the first time, a detailed investigation has been performed on heavily Fe-doped GaN structures to explore the 3D growth mode for further enhancing buffer resistivity. The effect of SiN_x_ nano-mask, developed previously by our group [6], on Fe diffusion and buffer leakage-current was also studied. Furthermore, the C-doped GaN layer was employed in the HEMT structure to restrict Fe diffusion by the existence of compressive stress. This further increased buffer V_BD_.

## 2. Materials and Methods

**Figure 1:**
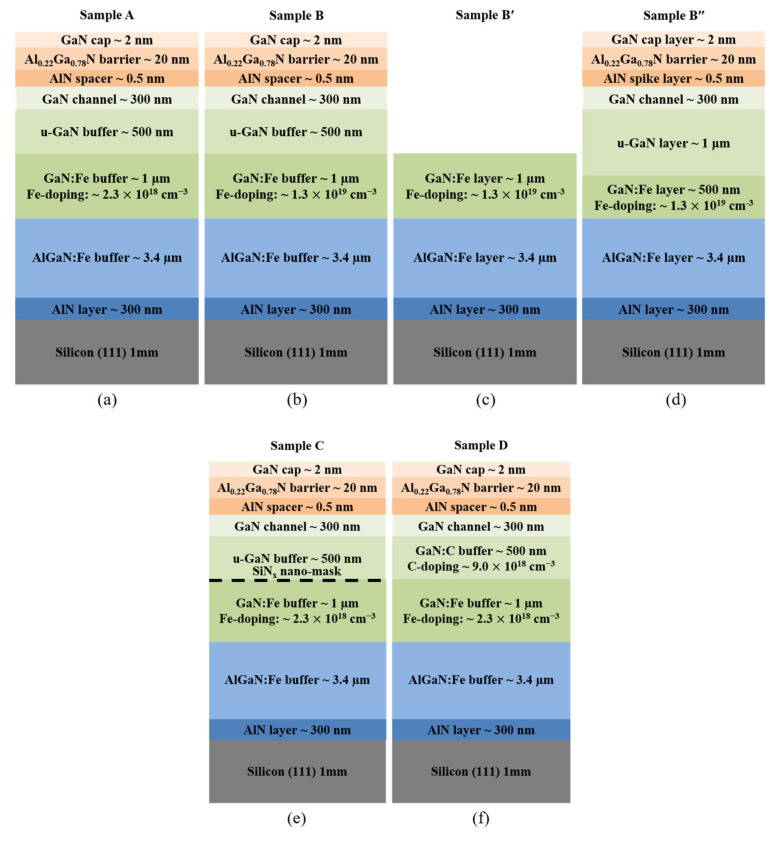
Schematic structures of Fe-doped GaN HEMT: (**a**) Sample A with Fe concentration 2.3 × 10^18^ cm^−3^ in the GaN:Fe buffer layer; (**b**) Sample B is the same as sample A, but with higher Fe concentration 1.3 × 10^19^ cm^−3^; (**c**) Sample B′ is the same as Sample B, but without the top layers; (**d**) Sample B″ is the same as Sample B, but with optimized thickness of GaN:Fe and u-GaN layers; (**e**) Sample C is the same as Sample A, but with a SiN_x_ nano-mask; and (**f**) Sample D is the same as Sample A, but with carbon concentration 9.0 × 10^18^ cm^−3^ in the 500 nm LT GaN buffer layer.

The epitaxial structures of the Fe-doped GaN HEMT were grown on the Si (111) substrates of 6″ in diameter and 1 mm-thickness by metal organic chemical vapor deposition (MOCVD) system (Aixtron G4, Herzogenrath, Germany). The trimethylgallium (TMGa), trimethylaluminum (TMAl), ammonia (NH_3_), Ferrocene (Cp_2_Fe), and CBrCl_3_ precursors (PentaPro Materials Inc., Tainan, Taiwan) were used as conventional sources for growing the AlN, AlGaN, GaN, Fe-doped GaN, and C-doped GaN layers. As shown in Figure 1, Samples A, B, B′, and B″ were used to study the effect of heavily Fe-doped GaN on the 3D growth mode. In addition, Samples A, B″, C, and D were employed to confirm the influence of different structures on Fe diffusion and V_BD_. All samples, except Sample B′, have the same total thickness of about 5.5 μm. Sample A is a control sample, which consists of 8 layers: (1) 300 nm AlN nucleation layer grown at 1040 °C and 50 mbar, (2) 3.4 μm high resistive AlGaN:Fe buffer layer grown at 1020 °C and 50 mbar, (3) 1.0 μm GaN:Fe buffer layer with Fe concentration of 2.3 × 10^18^ cm^−3^ grown at 1000 °C and 200 mbar, (4) 500 nm un-doped GaN (u-GaN) low temperature (LT) buffer layer grown at 990 °C and 200 mbar, (5) 300 nm GaN high temperature (HT) channel layer grown at 1020 °C and 200 mbar, (6) 0.5 nm AlN spacer grown at 1025 °C and 50 mbar, (7) 20 nm Al_0_._22_Ga_0_._78_N barrier layer grown at 1025 °C and 50 mbar, and (8) 2 nm GaN capping layer grown at 1025 °C and 50 mbar. In sample B, the 1 μm thick GaN:Fe layers were grown with heavy Fe concentrations of 1.3 × 10^19^ cm^−3^ to study the effect of Fe nano-mask on the 3D growth mode. To further explore the 2D to 3D growth mechanism, sample B′ was grown under the same growth conditions, but only until the formation of a 1 μm GaN:Fe buffer layer. Furthermore, the optimized structure of Sample B″ is different from sample B due to the presence of 500 nm GaN:Fe and 1 μm u-GaN layers. Sample C has an extra SiN_x_ nano-mask on the GaN:Fe buffer layer. In sample D, the u-GaN buffer layer was replaced by a C-doped GaN buffer layer with CBrCl_3_ dopant source. The Fe and C concentrations were determined by secondary ion mass spectroscopy (SIMS) measurement (CAMECA SAS, Gennevilliers, France). In situ reflectance was measured by the LayTec EpiCurve-TT (LayTec, Berlin, Germany) with a 633 nm light source to calculate the growth rate of epitaxial layers and investigate the transition from the 2D to the 3D growth mode. In order to investigate the effect of 3D growth on the surface morphology, the images of scanning electron microscopy (SEM, JSM7001F, JEOL, Tokyo, Japan) and the atomic force microscope (AFM, NT-MDT Spectrum Instruments, Moscow, Russia) were carefully observed. The crystalline quality of 3D growth samples was studied by X-ray diffraction (XRD, X’Pert Pro MRD, Malvern Panalytical, Almelo, The Netherlands) to evaluate the TD densities. In order to investigate the effect of 3D growth on electrical properties, the Van der Pauw–Hall measurement (HMS-3000, Ecopia Corporation, Anyang-City, South Korea) was performed at room temperature. The vertical current–voltage (I–V) characteristics of sample A, B″, C, and D were extracted to determine the vertical leakage current. Device fabrication with a patterning area (100 μm × 100 μm) was protected by a photoresist and then isolated by a reactive-ion etching (RIE) chamber using a plasma gas mixture of BCl_3_, Cl_2_, and Ar. In order to form the ohmic contacts, the Ti/Al/Ni/Au (25/130/25/90 nm) alloys have been deposited by electron beam evaporation, followed by rapid thermal annealing (RTA) at 875 °C for 30 s in the nitrogen environment.

## 3. Results and Discussion

**Figure 2:**
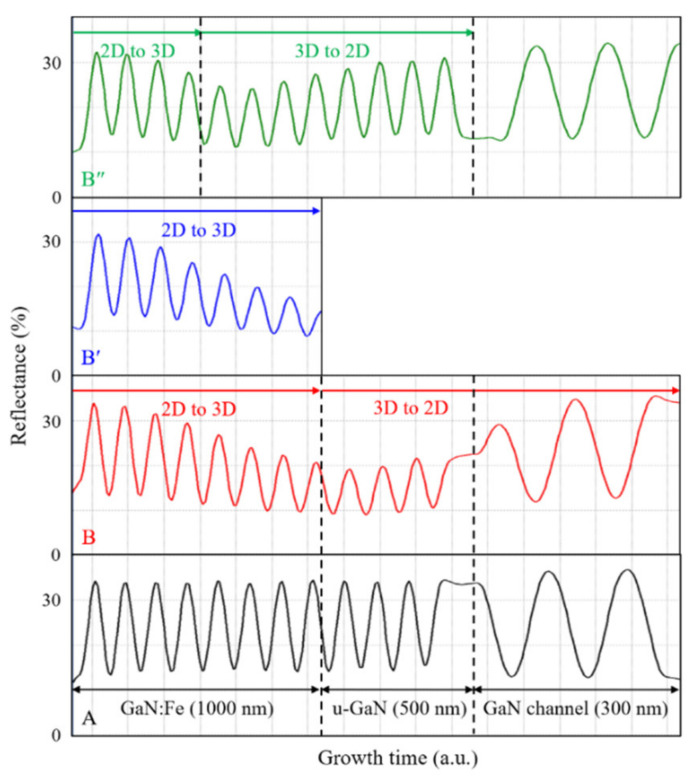
In situ reflectance monitoring on the surfaces of Samples A, B, B′, and B″.

**Figure 3:**
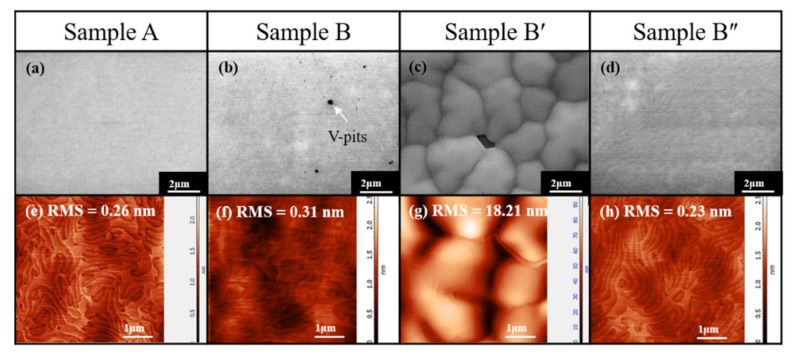
(**a**–**d**) SEM and (**e**–**h**) AFM images of the four Samples A, B, B′, and B″.

In order to investigate the effect of heavy Fe-doping on the 3D growth mode, the in situ reflectance measurements during the growth of the GaN:Fe layer to GaN channel layer for samples A, B, B′, and B″ were shown in Figure 2. The growth rate of these samples could be evaluated from the reflectance measurement. The growth rates of the GaN:Fe buffer, u-GaN LT buffer, and GaN HT channel layers are approximately 75 nm/min, 73 nm/min, and 30 nm/min, respectively. For Sample A, the reflectance intensity remains the same during the growth of the GaN:Fe and u-GaN buffer layers. It implies that the growth mode is 2D and the surface morphology is smooth during the GaN:Fe and u-GaN buffer growth. For Sample B, under the same growth conditions, the reflectance intensity of the GaN:Fe buffer layer gradually decreased with increasing growth time. This implies the transition of growth mode from 2D to 3D and results in increasing surface roughness. The heavy Fe-doping in Sample B results in a large amount of Fe segregation, which migrates to the sample surface during growth and forms Fe nano-masks to activate the growth of rough 3D islands [24,28]. Subsequently, the ferrocene delivery was terminated for the growth of the u-GaN LT buffer layer. At this stage, the growth mode started to vary progressively from 3D-island towards 2D-coalescence. However, at the end of u-GaN growth, the reflectance intensity did not fully recover to that of the 2D growth mode. The reflectance intensity of the following HT GaN channel layer rebounded rapidly and finally was almost restored to that of the 2D growth mode. The in situ reflectance measurement of Sample B′ is the same as that of the GaN:Fe layer of Sample B for illustrating the 2D to 3D growth. The above discussions were further supported by the surface morphology investigation in Figure 3. This result suggests that the heavy Fe-doping sample requires a thicker u-GaN buffer for fully recovering 2D growth before starting the growth of the HT GaN channel layer. Accordingly, Sample B″ was designed with 500 nm GaN:Fe and 1000 nm u-GaN buffer layers. The reflectance of B″ displays a relatively minor 3D growth mode compared with that of Sample B. It also demonstrates a better 3D to 2D growth mode transition during the thicker u-GaN overlayer growth. Current results illustrate that control of the relative thicknesses of GaN:Fe and u-GaN layers could manipulate the grain size of 3D-island growth and the time of full recovery.

The effect of heavy Fe doping on surface morphology was studied by SEM and AFM measurements, as shown in Figure 3. The surface morphology of the control sample A exhibited a smooth surface, as shown in Figure 3a. A root mean square (RMS) roughness of 0.26 nm is illustrated in Figure 3e, which agrees with the reflectance measurement of Figure 2. However, V-pits appeared on the surface of Sample B in Figure 3b, due to incomplete recovery from 3D to 2D growth. The 3D growth on the surface of the GaN:Fe layer is further verified by Figure 3c of Sample B′; the SEM image reveals numerous islands of about 3 μm in size after 1000 nm heavily Fe-doped GaN growth. Fe Segregations migrate to the surface to form nano-masks. The surface without Fe nano-masks has a higher growth rate for GaN:Fe with smaller Fe concentration compared with that of the GaN:Fe with larger Fe concentration grown on Fe nano-mask. As a result, 3D islands were observed in Figure 3c. Energy-dispersive X-ray spectroscopy (EDS) analysis on the islands and void found that the Fe content was about 1.13% in the void and was about 28 times higher than that on the islands of about 0.04%. The AFM image of Sample B′ in Figure 3g displays an RMS of 18.21 nm, corroborating the SEM result. Finally, the surface morphology of Sample B″ illustrates a smooth surface without V-pits and small roughness of 0.23 nm RMS, as shown in Figure 3d,h, respectively. This is due to the adjusted thickness of the GaN:Fe and u-GaN layers, which decreases the grain size of 3D islands and increases recovery duration to resume 2D-growth.

In order to study the effect of 3D growth on the crystalline quality of the GaN epilayers with different GaN:Fe conditions, HRXRD rocking curve measurements were performed. Both the mosaic model and Kaganer model could be used to determine the dislocation density [29,30,31,32]. In Table 1, the FWHM of GaN (002) and (102) planes (i.e., $\beta_{\left(002\right)}$ and $\beta_{\left(102\right)}$) can be analyzed with the mosaic model to identify the type of dislocations (screw or edge-type) by the following formulas [6]:

(1) $$D_{screw}=\frac{\beta_{\left(002\right)}^{2}}{4.35\times b_{screw}^{2}},\;\;b_{screw}=0.5185\;\mathrm{nm}$$

(2) $$D_{edge}=\frac{\beta_{\left(102\right)}^{2}-\beta_{\left(002\right)}^{2}}{4.35\times b_{edge}^{2}},\;\;b_{edge}=0.3189\;\mathrm{nm}$$

The screw-type TD densities (*D_screw_*) and edge-type TD densities (*D_edge_*) were calculated by $\beta_{\left(002\right)}$, $\beta_{\left(102\right)}$, and Burger vector length (*b_screw_* and *b_edge_*). The FWHM of GaN (002) plane corresponding to the screw-type TD densities showed similar results on these three samples. However, the FWHM of the GaN (102) plane was relatively sensitive to the 3D growth mode. The edge-type TD densities of Sample B decreased significantly by around 30%, from 2.47 × 10^9^ to 1.75 × 10^9^ cm^−2^, attributed to incomplete 3D growth to 2D-coalescence process. Apparently, the accumulation of extra Fe atoms as a mask partially impeded the propagation of TDs to terminate or bend at the 3D island-like GaN. Following the growth of GaN:Fe, the occurrence of the coalescence process in u-GaN resulted in the edge dislocations bending and being annihilated in the 3D to 2D lateral overgrowth layer. A similar result was also observed in Sample B″; the total TD densities also reduced from 2.81 × 10^9^ to 2.32 × 10^9^ cm^−2^ due to the transition of 2D-3D-2D growth by Fe nano-mask. Current investigation is similar to our previous publication, which reports the application of a SiN nano-mask to decrease dislocation density [6].

The effect of 3D growth on the transport properties of GaN HEMT structures was studied by van der Pauw–Hall measurements on Samples A, B, and B″, and the results were summarized in Table 1. The control Sample A has a sheet carrier concentration (Ns) and mobility of 6.5 × 10^12^ cm^−2^ and 1580 cm^2^/V·s, respectively. Sample B revealed that both Ns and mobility degraded significantly, indicating that the appearance of V-pits could cause not only the rough interface, but also a trap center at the two-dimensional electron gas (2DEG) channel [33,34,35]. This result is in agreement with our previous research; it illustrates that the decreased Ns and mobility were caused by the incomplete coalescence process [6]. Finally, lower Rs and higher Ns were observed in the optimized Sample B″, which implies that the 3D growth mode from Fe nano-mask can effectively decrease TD densities under the conditions of a full recovery of 2D growth in the LT u-GaN layer and good crystal quality of the HT GaN channel layer.

**Figure 4:**
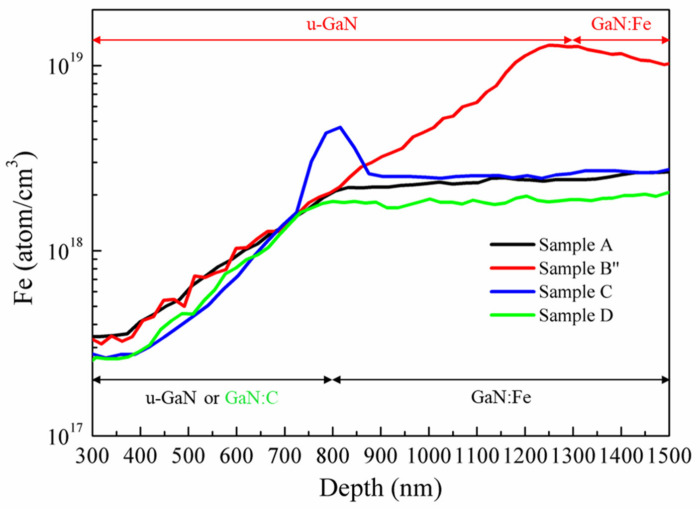
Fe concentration profile measured by SIMS for four different structures.

The effect of different sample structures in A, B″, C, and D on Fe diffusion towards the u-GaN overlayer is shown in Figure 4, the profile of Fe diffusion measured by SIMS. This is consistent with the result of the references [26,36]; Fe atoms incorporated into the u-GaN layer via Fe segregation from the growth surface. The diffusion slope in the u-GaN buffer layer reflects the Fe concentration gradient. If the Fe diffusion follows Fick’s first law, then:

(3) $$J=-D\frac{\partial \rho }{\partial x}$$

where J is diffusion flux, ρ is the concentration of Fe, ∂ρ/∂x is the Fe concentration gradient, and D is the diffusion constant. Suppose that D is the same for all samples. Then, the same slope of about 0.0017 for Samples A and B″ demonstrates that the Fe diffusion flux is not relevant to the Fe doping concentration, 3D growth mode, and the edge-type TD densities. It is interesting to note that Mg diffusion along the edge- and mixed-type TDs was evidenced [37]. However, our current study reveals that the edge-type TDs densities are not the key factor on Fe diffusion. In Sample C, the Fe diffusion slope of u-GaN buffer was increased by 41% to 0.0024 and also showed a pronounced increment in slope near the SiN_x_ nano-mask. This indicates that the SiNx nano-mask could not only compensate with Fe atoms on the Ga site, but also block the diffusion of Fe atoms as a filter function. For Sample D, the GaN:C layer also showed a larger Fe diffusion slope of 0.0021. This indicates that the GaN:C acts as an effective layer to suppress Fe diffusion by the induced compressive strain resulting from the replacement of the Ga atom by the smaller C atom [27]. We conclude that the major factors for effectively suppressing Fe diffusion are compressive stress and the SiN_x_ nano-mask. The effect of 3D growth and the Fe nano-mask plays a minor role for suppressing Fe diffusion as extracted from the SIMS results of Samples A and B″. Although the transition of 3D-to-2D growth mode could not effectively suppress the Fe diffusion, it significantly improves crystalline quality and 2DEG performance.

**Figure 5:**
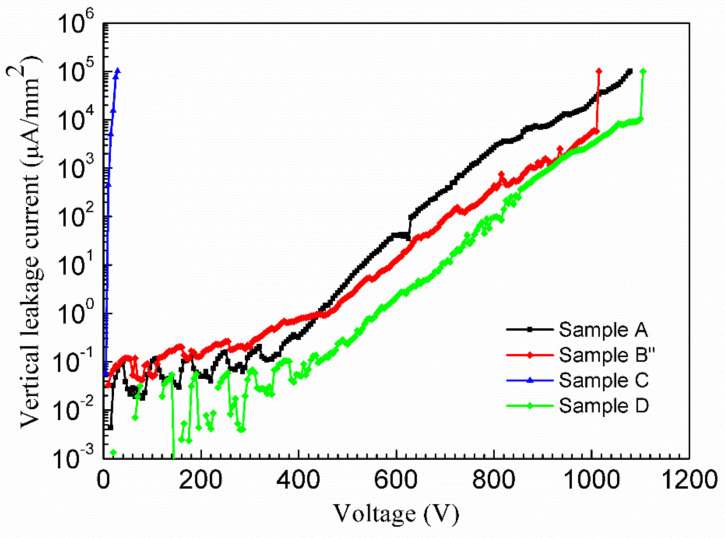
Vertical leakage current performance of the four samples at room temperatures.

Finally, vertical leakage current measurements were performed at room temperature on the four HEMT structures through the grounded substrate, and the ohmic contact pattern was swept from 0 V to the breakdown voltage, as shown in Figure 5. The breakdown voltage is determined by the vertical leakage current reaching 1 mA. The vertical leakage current versus applied voltage curves of Samples A, B″, and D exhibited very good electrical characteristics; they showed breakdown voltages of 1075 V, 1010 V, and 1100 V, respectively. However, for Sample C with the insertion of a SiN_x_ nano-mask, the vertical leakage current increased significantly at low applied voltage. This implies that the donor-like SiN_x_ nano-mask could form high densities of free electrons due to Si-doping into the GaN layer. Therefore, the SiN_x_ nano-mask growth technique is a good method to reduce the dislocation density [6], but it has a problem for high-power application. Moreover, the vertical leakage current of Sample B″ is reduced by an order of magnitude for an applied voltage of 1000 V in comparison to that of Sample A, despite the optimized Sample B″ having a thinner GaN:Fe insulating layer. This is attributed to the effective enhancing of the buffer insulation and decreasing TD density by the *p*-type Fe nano-mask. In addition, Sample D exhibited the best performance on vertical leakage current among all the samples, as it consists of both GaN:Fe and GaN:C buffer layers. Investigation on the structures with the presence of both Fe- and C-doping in buffer is worth performing, and how it impacts the buffer resistivity as well as GaN power device performance has not been understood yet. Herein, for the first time, we demonstrated the effect of 3D growth with a *p*-type Fe nano-mask on buffer leakage current to replace the *n*-type SiN_x_ nano-mask for high-power HEMT application.

## 4. Conclusions

The effect of *p*-type Fe nano-mask on 3D island growth was studied for the application in high power GaN HEMT. The SEM image verified that Fe segregation generates a growth mask to initiate a 3D growth mode in the GaN:Fe buffer layer. Better crystal quality and 2DEG properties were demonstrated by induced 3D growth technology. However, 3D growth and dislocation bending by *p*-type Fe nano-mask could not affect Fe diffusion in the u-GaN layer. For the first time, we discovered that the Fe nano-mask 3D growth technique can decrease vertical leakage current by an order of magnitude at a voltage 1000 V due to the decrease in buffer conductivity and TD density. The samples of both GaN:Fe and GaN:C buffer layers exhibited the best performance in suppression of Fe-diffusion and buffer leakage-current. These vertical I–V characteristics evidence the important effect of highly insulating buffer and low dislocation density on vertical current leakage. It is highly beneficial for applications in high power GaN HEMT.

## Figures and Tables

**Table 1:** Dependence of Hall, XRD measurements, and TD densities on different GaN:Fe conditions.

Sample ID	Sheet Resistance (ohm/sq)	Sheet Carrier Concentration (cm^−2^)	Mobility (cm^2^/V·s)	XRD (arcsec)		TD Densities (cm^−^^2^)	
				(002)	(102)	Screw-Type	Edge-Type
A	610	6.5 × 10^12^	1580	407.4	794.8	3.34 × 10^8^	2.47 × 10^9^
B	1227	5.5 × 10^12^	933	403.2	700.8	3.27 × 10^8^	1.75 × 10^9^
B″	516	7.8 × 10^12^	1556	394.2	730.2	3.12 × 10^8^	2.01 × 10^9^

## Data Availability

Data are contained within the article.
